# Strongylus Contortus in a Herd of Angora Goats

**Published:** 1900-07

**Authors:** H. E. Titus

**Affiliations:** Maxwell, Iowa


### STRONGYLUS CONTORTUS IN A HERD OF ANGORA GOATS.

By H. E. Titus, D.V.M.,
MAXWELL, IOWA.

In reporting this case it is not with the idea that it is a rare one, but simply my first experience in dealing with the *strongylus contortus* in the Angora goat.

This herd, some two hundred in number, ranged in age from one to five years, the yearlings coming from Kansas, and the older ones from Texas. I was called first on December 11th to account for three deaths which had occurred some days prior to that time. On visiting the herd I found two yearlings sick and unable to stand, and perhaps a dozen yearlings moping around, some few of them showing a slight diarrhœa.

It was deemed advisable to destroy and post-mortem one of the worst cases. The post-mortem revealed the presence of the small thread-like parasite, apparently making up the greater portion of the contents of the fourth stomach.

I at once began preparation for treating the entire herd. This consisted of drenching each individual one by one with two to four ounces of an emulsion of oil of turpentine and milk, one pail of turpentine to sixteen of the milk. This treatment was repeated at intervals of four days until four doses had been administered. Those showing intestinal disturbanccs, but yet able to be on their feet, were treated by the following prescription: Nucis vomica., ℥ij; gentian pulv., ℥vj; sodii bicarb, ℥iv; misc. This to be divided into forty-two powders, and one-half powder administered with gruel twice per day to each animal.

There has been but one death since treatment began, and the remainder of the herd is gradually improving in general appearance.

---

Pennsylvania and Maryland State Veterinary Examining Boards conducted examinations in June at Philadelphia and Baltimore.

